# RNA-seq transcriptome analysis of formalin fixed, paraffin-embedded canine meningioma

**DOI:** 10.1371/journal.pone.0187150

**Published:** 2017-10-26

**Authors:** Jennifer K. Grenier, Polly A. Foureman, Erica A. Sloma, Andrew D. Miller

**Affiliations:** 1 Department of Biomedical Sciences, Cornell University College of Veterinary Medicine, Ithaca, New York, United States of America; 2 Division of Biological Sciences, Chandler-Gilbert Community College, Chandler, Arizona, United States of America; Colorado State University, UNITED STATES

## Abstract

Meningiomas are the most commonly reported primary intracranial tumor in dogs and humans and between the two species there are similarities in histology and biologic behavior. Due to these similarities, dogs have been proposed as models for meningioma pathobiology. However, little is known about specific pathways and individual genes that are involved in the development and progression of canine meningioma. In addition, studies are lacking that utilize RNAseq to characterize gene expression in clinical cases of canine meningioma. The primary objective of this study was to develop a technique for which high quality RNA can be extracted from formalin-fixed, paraffin embedded tissue and then used for transcriptome analysis to determine patterns of gene expression. RNA was extracted from thirteen canine meningiomas–eleven from formalin fixed and two flash-frozen. These represented six grade I and seven grade II meningiomas based on the World Health Organization classification system for human meningioma. RNA was also extracted from fresh frozen leptomeninges from three control dogs for comparison. RNAseq libraries made from formalin fixed tissue were of sufficient quality to successfully identify 125 significantly differentially expressed genes, the majority of which were related to oncogenic processes. Twelve genes (AQP1, BMPER, FBLN2, FRZB, MEDAG, MYC, PAMR1, PDGFRL, PDPN, PECAM1, PERP, ZC2HC1C) were validated using qPCR. Among the differentially expressed genes were oncogenes, tumor suppressors, transcription factors, VEGF-related genes, and members of the WNT pathway. Our work demonstrates that RNA of sufficient quality can be extracted from FFPE canine meningioma samples to provide biologically relevant transcriptome analyses using a next-generation sequencing technique, such as RNA-seq.

## Introduction

Meningiomas are the most commonly reported intracranial neoplasm in dogs [1,2], cats [3], and humans [4]. They arise from arachnoid cap cells that line arachnoid villi and are found in the middle layer of the meninges [5]. The incidence of primary brain cancer in the canine population is difficult to establish and estimates come from series of necropsied dogs. One recently reported series [2], found 435 cases of brain cancer among 7969 (5.5%) one-year-old or greater necropsied dogs in the years from 1986 to 2010. Of the 435 intracranial neoplasms in that series, 227 were primary intracranial tumors, and of these, roughly half were meningiomas (117/227) [2].

Dogs have been proposed as a large animal model for human neoplasia, not only for understanding the pathology of cancer, but also as a surrogate in drug development and treatment. Cancer arises spontaneously in pet dogs, often at a rate comparable to that in humans, but on a shorter time scale. Pet dogs share a common environment with their human companions and may be useful as sentinels for carcinogens in the environment. In addition, because of their breeding history, while there is genetic heterogeneity comparable to that in humans, particular breeds have less genetic heterogeneity and may prove to have a superior background for identifying cancer related genes [6–9].

In the dog, meningiomas arise most commonly in the brain and less commonly in the spinal cord and retrobulbar space originating from the optic nerve [1]. Meningiomas are highly variable histopathologically and tend to be slow-growing cancers in veterinary and human patients. Canine meningiomas recapitulate the majority of the histologic subtypes reported in humans, with the low-grade meningothelial and transitional subtypes being most common in dogs [10,11]. The malignant potential for canine meningiomas is not fully understood, nor is it clear that the human World Health Organization (WHO) grading scheme is applicable to the dog even though it is widely applied to canine cases of meningioma due to lack of an accepted equivalent canine grading scheme [12]. It is clear that histology alone is not a reliable predictor of the biological behavior of canine meningiomas.

While there are many studies of protein expression and transcriptome analysis of human meningiomas, identification of a set of reliable factors related to cancer behavior and prognosis has proved elusive, perhaps because of the heterogeneous nature of meningiomas and due to a lack of consistency in experimental design. Most studies compare differentially expressed (DE) genes in one tumor grade to another, some compare meningiomas to normal brain tissue, and only a handful of studies compare DE in meningiomas to meningeal, or specifically arachnoid, tissue [13]. Several DE changes are widely accepted for human meningiomas, with association to genetic alterations of the type 2 neurofibromatosis gene (NF2; chromosome 22q) being the best documented. Signaling pathways whose dysregulation are linked to progression, tumorigenesis and proliferation include RB/p53 pathway, the WNT/β-catenin pathway, Notch pathway, P13K/Akt/MAPK pathway, and the Hedgehog pathway. In addition, recurrent chromosome alterations have been identified, including 1p, 6q, 9p, 10, 14q, 17, 18p, and 22q [14–17].

Due to an overreliance on histologic appearance and immunohistochemical alterations in canine meningioma, there is limited gene expression data published for canine meningiomas. The lack of robust molecular data in canine meningioma is partly due to the difficulties in obtaining fresh tumor tissue from surgically resected cases of canine meningioma. This is predominately related to the lack of access to rapid freezing modalities (like liquid nitrogen) in most private veterinary practices. The majority of surgically excised canine meningiomas are submitted to diagnostic laboratories as formalin-fixed tissue and being able to utilize this tissue for more than basic histologic and immunohistochemical analysis would be ideal to expand the scope and sample availability to study the gene expression patterns characteristic of meningioma, in order to better understand this disease.

Given the lack of global gene expression data in canine meningioma and the similar biological behavior, histology, and immunophenotype as human meningiomas, the goal of the current study was two-fold. Initial work demonstrated a reliable method to generate gene expression data from formalin-fixed, paraffin-embedded (FFPE) cases of canine meningioma. Once a reproducible and reliable method was developed, in-depth gene expression analysis was performed on a small cohort of canine meningioma cases. While the sample numbers presented herein are not large enough to generate a highly statistically powered analysis of canine meningioma, the data do reveal genes of interest that are candidates for further investigation in future, larger studies. Until recently, transcriptome evaluation of differentially expressed genes have required tissue that was flash frozen or preserved in a special solution with an RNase inhibitor (e.g. RNA*later*®) in order to provide RNA of adequate quality for gene expression analysis. Because most current and archived veterinary samples are FFPE, it would greatly expand the availability of material for analysis if these could be used as a source of RNA. Recent studies have shown that reliable RNA can be obtained from FFPE samples as much as 40 years old [18] and analyzed using an Affymetrix microarray platform [19] or using transcriptome sequencing such as RNAseq [20]. We report here a proof of concept study with the extraction of sufficient quality RNA from FFPE archival samples and demonstrate their utility in transcriptome analysis using RNAseq to evaluate differential gene expression in canine meningioma.

## Materials and methods

### Samples and patient population

Samples were obtained from biopsy specimens submitted to the New York State Animal Health Diagnostic Center, Cornell University College of Veterinary Medicine, Section of Anatomic Pathology over a ten year period from 2004 to 2014 from referring veterinarians as part of the surgical biopsy service. All meningioma specimens were submitted in 10% neutral buffered formalin and were embedded in paraffin wax within 24 hours of arrival. For 2 cases, a portion of the biopsy was flash-frozen for RNA isolation (P7, P12). Diagnoses were made based on hematoxylin and eosin stained sections reviewed by a board-certified veterinary pathologist (ADM). The histologic features and grade were assigned based on the criteria set forth in the WHO Classification of Tumors of the Central Nervous System. Control samples of leptomeninges were flash-frozen after collection from three healthy research beagle dogs shortly after euthanasia for an unrelated project. The three research beagles were cared for in accordance with the National Research Council’s Guide for the Care and Use of Laboratory Animals (8^th^ edition, 2011) and the standards of the Cornell University Institutional Animal Care and Use Committee.

Thirteen dogs diagnosed with a meningioma were included (Table 1). The average age was 11.5 (range: 7 to 14 years old). There were nine females and four males which included Labrador retrievers (three) and one each of standard poodle, poodle (unspecified), Scottish terrier, husky, keeshond, dachshund, Shetland sheepdog, corgi, Chihuahua, and mixed breed dog.

**Table 1:** Clinicopathologic features of canine meningioma cases.

Case Number	Breed	Sex	Age (years)	Diagnosis
1	Scottish Terrier	FS^a^	9	Transitional; grade I
2	Dachshund	FS	14	Meningothelial; grade I
3	Chihuahua	FS	7	Transitional; grade I
4	Keeshond	MC^b^	10	Transitional; grade I
5	Siberian husky	FS	12	Transitional; grade I
6	Standard poodle	FS	9	Transitional; grade I
7*	Poodle	MC	14	Atypical; grade II
8	Shetland sheepdog	FS	13	Atypical; grade II
9	Corgi	MC	11	Atypical; grade II
10	Labrador retriever	MC	12	Atypical; grade II
11	Mixed breed	FS	13	Atypical; grade II
12*	Labrador retriever	FS	13	Atypical; grade II
13	Labrador retriever	FS	13	Atypical; grade II

Breed, sex, age, and histopathologic diagnosis for cases of canine meningioma.

^a^Female spayed

^b^Male castrated

*RNA isolated from fresh-frozen meningioma tissue

Meningioma location was described for 12 of the 13 tumors. 5/12 (42%) were olfactory, 4/12 (33%) were located in the anterior dorsal aspect of the cerebrum (frontal and/or parietal lobe), one was cerebellar (8%), and two (17%) were located in the spinal column (one in the lumbar region; the other was not specified). The normal, control meninges were collected over the frontal, temporal, and occipital cortices.

### RNA purification

For FFPE samples, two 15μm scrolls were cut from each paraffin block. The scrolls were stored at -80°C. RNA from FFPE samples was purified using the Agencourt FormaPure kit (Beckman Coulter). Samples were heated in buffer to release the tissue from paraffin and to reverse crosslinking. This was performed in lysis buffer (Beckman Coulter) at 70° for one hour. Total nucleic acids were freed from the tissue by protease K digestion (Beckman Coulter) at 55° for one hour. Samples were transferred to a 96-well plate that was compatible with the BioMek 4000 Automated Liquid Handling System (Beckman Coulter). The automated system binds nucleic acids to carboxyl-coated magnetic beads, immobilizes the bead-bound nucleic acids using a magnet provided with the system, treats the samples with DNase to remove genomic DNA, and then washes with alcohol solutions. This process begins with the addition of a binding buffer to the samples, then beads, suspended in isopropanol, are added. The samples are incubated at 55° to facilitate binding. After incubation the samples are transferred to a magnetic plate (Beckman Coulter). Then the samples are washed with 85% ethanol and allowed to dry (Beckman Coulter). Next, DNase is added and incubated at 37° for 15 minutes. The samples are re-bound to the beads in wash buffer (Beckman Coulter). Using the magnetic plate, the wash buffer is removed and samples are washed with 90% isopropanol then with 85% ethanol. Finally the samples are air dried and eluted in 70μL of RNase free water. The standard protocol was modified by increasing the ethanol concentration to 85% from 70% to improve the yield of small RNAs. RNA was eluted off the beads using nuclease-free water. For fresh-frozen samples, RNA was purified using Trizol (Invitrogen) according to the manufacture’s protocol.

The concentration and purity of the samples was assessed by spectrophotometry (Nanodrop; ThermoScientific) and RNA integrity was quantified on a Bioanalyzer (Agilent Technologies) or a Fragment Analyzer (Advanced Analytical). RNA integrity was variable across samples, but showed clear RNA degradation, as expected for FFPE samples, with potential genomic DNA (high molecular weight material) remaining in some cases. Samples with high molecular weight material were treated with RapidOUT DNAse (ThermoFisher Scientific) and the buffer exchanged over a Micro Bio-Spin 30 column (BioRad) and re-quantified by spectrophotometry prior to rRNA subtraction. Ribosomal RNA was subtracted by hybridization from 1–2.5ug total RNA per sample using the RiboZero Magnetic Gold H/M/R Kit (Illumina). Following cleanup by precipitation, rRNA-subtracted samples were quantified with Qubit 2.0 (RNA HS kit; Thermo Fisher).

### Illumina library preparation and sequencing

TruSeq-barcoded RNAseq libraries were generated with the NEBNext Ultra Directional RNA Library Prep Kit (New England Biolabs) using 50-100ng rRNA-subtracted RNA. The RNA fragmentation time was adjusted between 0–15 minutes per sample to account for the degree of fragmentation determined by the RNA integrity check. Each library was quantified with Qubit 2.0 (dsDNA HS kit; Thermo Fisher) and the size distribution was determined with a Fragment Analyzer (Advanced Analytical) prior to pooling. All libraries had a similar size distribution, typically with a size peak between 300-400bp. Libraries were sequenced on an Illumina HiSeq2500. At least 20M single-end 100bp reads were generated per library.

### Analysis

Raw reads were trimmed for low quality and adaptor sequences and filtered for minimum length with cutadapt software (parameters: -m 20 -q 20 -a AGATCGGAAGAGCACACGTCTGAACTCCAG—match-read-wildcards) [21]. Because residual rRNA-matching reads can affect RNAseq normalization and small RNAs are not well quantified with RNAseq, reads matching rRNA and small RNAs (e.g. snRNAs, snoRNAs, microRNAs) were removed with bowtie2 v2.2 (parameters: default end-to-end mapping options, using—un-gz to retain non-matching reads) [22]. The remaining reads were mapped to the reference genome/transcriptome (*Ensembl CanFam3)* using tophat v2.0 (parameters:—library-type = fr-firststrand—no-novel-juncs -G <Ensembl_CanFam3_genes.gtf>) [23]. Differential gene expression was analyzed with cufflinks v2.2 [24]. JMP 11 (SAS) was used for principal components analysis, using log2 transformed FPKM values for the 8,930 genes with detectable and variable gene expression across the dataset. For the 125 genes found to be differentially expressed between meningioma and control samples, JMP 11 was used for unsupervised 2-way hierarchical clustering and Panther (http://www.pantherdb.org/; statistical overrepresentation test with default settings) was used to determine enrichment of GO terms [25]. RSeQC v2.6 was used for gene body coverage analysis [26]. After ‘humanizing’ the canine dataset using Biomart (Ensembl) one-to-one orthology assignments for protein-coding genes, gene set enrichment analysis (GSEA) was used for gene set enrichment analysis of Hallmark gene sets from MSigDB (GSEA pre-ranked on log2-fold change values with “classic” enrichment statistic) [27, 28]. Gene expression data are available at the Gene Expression Omnibus (GEO) [at NCBI], accession number GSE95048.

### Real-time RT-PCR validation

The levels of expression of a subset of differentially expressed genes were validated using real-time reverse transcription polymerase chain reaction (qPCR). cDNA was synthesized from 500ng of the original total RNA sample or 50ng of rRNA-depleted RNA (when original total RNA was no longer available) using M-MuLV reverse transcriptase (Sigma), buffer, and a mix of oligo-dT(20) and random 9-mer oligo (IDT) primers (2uM and 1uM respectively) in a 20ul reaction volume. RNA was denatured at 65°C for 1 minute prior to addition of reverse transcription reaction reagents, and the reaction was incubated at 37°C for 1 hour followed by 10 minutes at 85°C to inactive the reverse transcriptase. All cDNA reactions were diluted 20-fold with water prior to use in qPCR (such that 5ul used in qPCR is equivalent to 0.25ul original cDNA reaction).

Primer pairs (Table 2) were designed with Primer-BLAST (NCBI) to extend across an exon boundary in all cases to minimize amplification of residual contaminating genomic DNA and allow identification of alternate amplicons with melt curve analysis. Endogenous control primers were chosen based on published results of reference genes [29,30] Each primer pair was validated using a standard curve of six four-fold serial dilutions of a representative sample of pooled cDNA. A ‘No-RT’ control containing RNA but lacking M-MuLV enzyme and one ‘no template’ control lacking any cDNA sample was included for each primer pair standard curve validation. Primer pairs that did not generate signal in <35 cycles or that exhibited non-quantitative performance (i.e. ≠2-cycle shifts for 4-fold dilution series), non-specific signal in negative controls, or variable amplicon identities as determined by melt curve analysis were excluded. All of the primer pairs in Table 2 passed validation by standard curve testing.

**Table 2:** Primer pairs used in the qPCR analysis.

Gene	Forward (5´ → 3´)	Reverse (5´ → 3´)	Amplicon Length (base pairs)
AQP1	CCTTCCGGACAACTCCCTCG	AGCCCTGACCGGAGTTCAC	62
BMPER	TGTGTTCTACGTCAGTGCCAG	TGTGTTCTACGTCAGTGCCAG	60
FBLN2	ATCATGGCGGATGGTGTGTC	CCCATGAGGCACTCGTCTTG	50
FRZB	AACGGAAACTGTAGAGGGACC	GACAGGCTTACATTTGCAGCG	51
MEDAG	TACCGCCTCAGCAGCTACATC	AATCGCAGTAGTTGGTCAGTTCC	55
MYC	CCTCCGGAGAGTGGAAACCC	GCTGACGTTGAGAGGCATCG	49
PAMR1	AGTCCTTCCCATGCAGGTTC	AGAGCTGGTGTAACGGTGTC	50
PDGFRL	AAGTACCAGCTGCTCTACGTG	AGATGGTTGTTGATGGAGGGC	55
PDPN	AGAGCACCACAACCTTGAATG	AACCGTTGTCTCGGTGTCTTC	74
PECAM1	TGACCTCACCTGAGCCTTAC	GTCAAGGGAGCCTTCCGTTC	45
PERP	CGTCTTCCTGAGAGTGATTGGAG	CCAGGGAGATGATCTGGAATAC	65
ZC2HC1C	AGAGCAGTACCTGAACTGGAAG	TTGCTCTTCTGAGGAGGTTCAG	63
B2M^a^	TCCTCATCCTCCTCGCT	TTCTCTGCTGGGTGTCG	85
GUSB^a^	AGACGCTTCCAAGTACCCC	AGGTGTGGTGTAGAGGAGCAC	103
RPL13A^a^	GCCGGAAGGTTGTAGTCGT	GGAGGAAGGCCAGGTAATTC	87

Genes, primer pairs, and amplicon size for qPCR analysis.

^a^Endogenous control genes

qPCR reactions were prepared in 10 uL reaction volumes in an optically clear 384-well PCR plate with seal (Roche) using the LightCycler 480 SYBR Green I Master Mix (Roche) with 0.25 uM primers and 5 uL pre-diluted sample cDNA. All reactions were performed in triplicate using a Roche LightCycler 480 instrument. Cycles were: initial incubation 5 minutes at 95^o^ C; followed by 45 cycles of 30 seconds at 95^o^ C; 30 seconds at 60^o^ C; 10 seconds at 72^o^ C; and final a melt curve with a ramp from 60^o^ C to 95^o^ C at 2^o^ C per second. Melt curve analysis was used to identify and exclude reactions with alternative amplicons. For relative quantification estimates for each target gene, the ΔΔ Ct value [ΔCt_SAMPLE_ - ΔCt_REF_] was calculated for each sample, where ΔCt_SAMPLE_ = average (target gene Ct)—average (all endogenous control Ct) and ΔCt_REF_ was defined as the average ΔCt_SAMPLE_ for the normal samples. The normalized relative amount of the target gene is 2^-ΔΔCt^ [31]. REST 2009 v2.0.13 was used to determine whether the qPCR data showed a significant difference in target gene expression between patient and control samples; only patient samples with >2-fold change in the RNAseq data were used, thus excluding patients with unaltered gene expression relative to controls [32].

## Results

### Gene expression profiling

RNA was initially extracted and subjected to RNAseq analysis for twenty-four meningioma samples and three control meninges. However, of these twenty-four cases, several samples were omitted because they failed quality control checks during RNAseq library prep and analysis (low genome mapping rate <80% [four cases], outliers in preliminary principal components analyses [five cases]; data not shown) or upon further investigation were not consistent with a meningioma (two cases). These last two cases that were omitted had histologic features comparable with malignant meningioma; however, were noted to have vastly different expression profiles than all other samples. Genes highly expressed in only these samples included many genes associated with melanocytic differentiation. Additional information on these cases acquired from the referring veterinarians, in addition to immunohistochemical confirmation of melanocytic differentiation, led to the revised diagnosis of metastatic melanoma, rather than meningioma. These quality control filters were important to eliminate poor quality or non-meningioma samples and to focus the final analysis to the highest quality RNAseq data with the best biological signal for meningioma profiling.

On average, over 30 million (M) reads were generated per sample (minimum 20M reads; S1 Table). Typically, 10–20% of reads matched ribosomal RNA and were removed prior to analysis with Tophat. In addition to rRNA-matching reads, any reads that match annotated microRNAs, snRNAs, snoRNAs, or other short (<250nt) noncoding RNAs were removed because these genes are not well quantified with RNAseq libraries made with random-primed double-stranded cDNA. After read trimming and filtering, genome mapping rates exceeded 80% (average 88%) with ~5M reads matching the annotated transcriptome on average (S1 Table). Analysis of gene body coverage indicated consistent coverage with minimal 3’ bias for all samples (S1 Fig).

**Fig 1:**
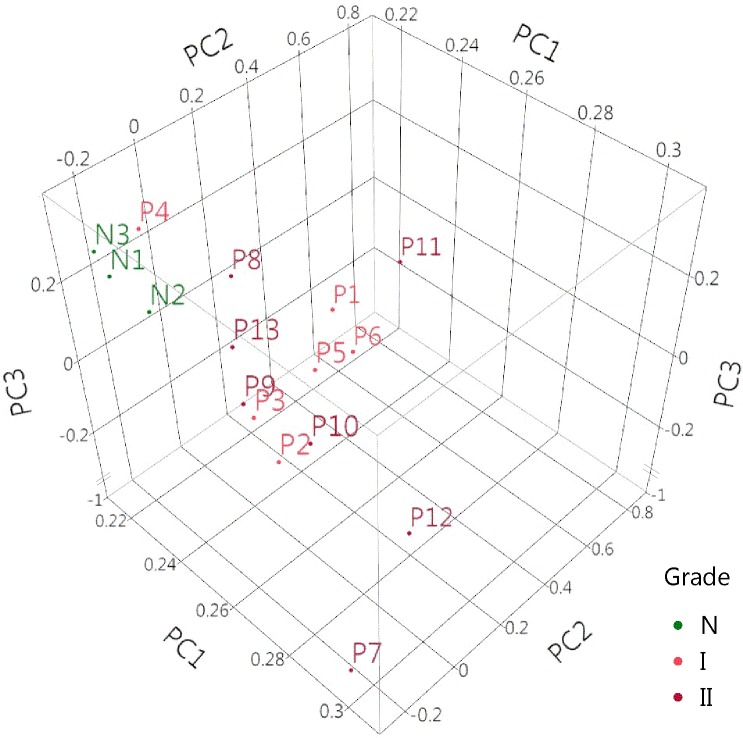
Principal components analysis. Gene expression profiles were performed on normal canine meninges and canine meningioma samples and subject to principal components analysis. Eigenvalues: PC1 = 53.4 (75%), PC2 = 3.0 (4.3%), PC3 = 2.4 (3.4%).

Principal components analysis of the thirteen primary meningioma samples and three control meninges (Fig 1) showed close grouping of the normal cohort samples, with the meningiomas distributed more widely, indicating dissimilar gene expression profiles. The fresh-frozen samples (P7, P12, and all 3 normal samples) did not cluster together, indicating that the sample collection and preparation did not influence the expression profiling data.

**Fig 2:**
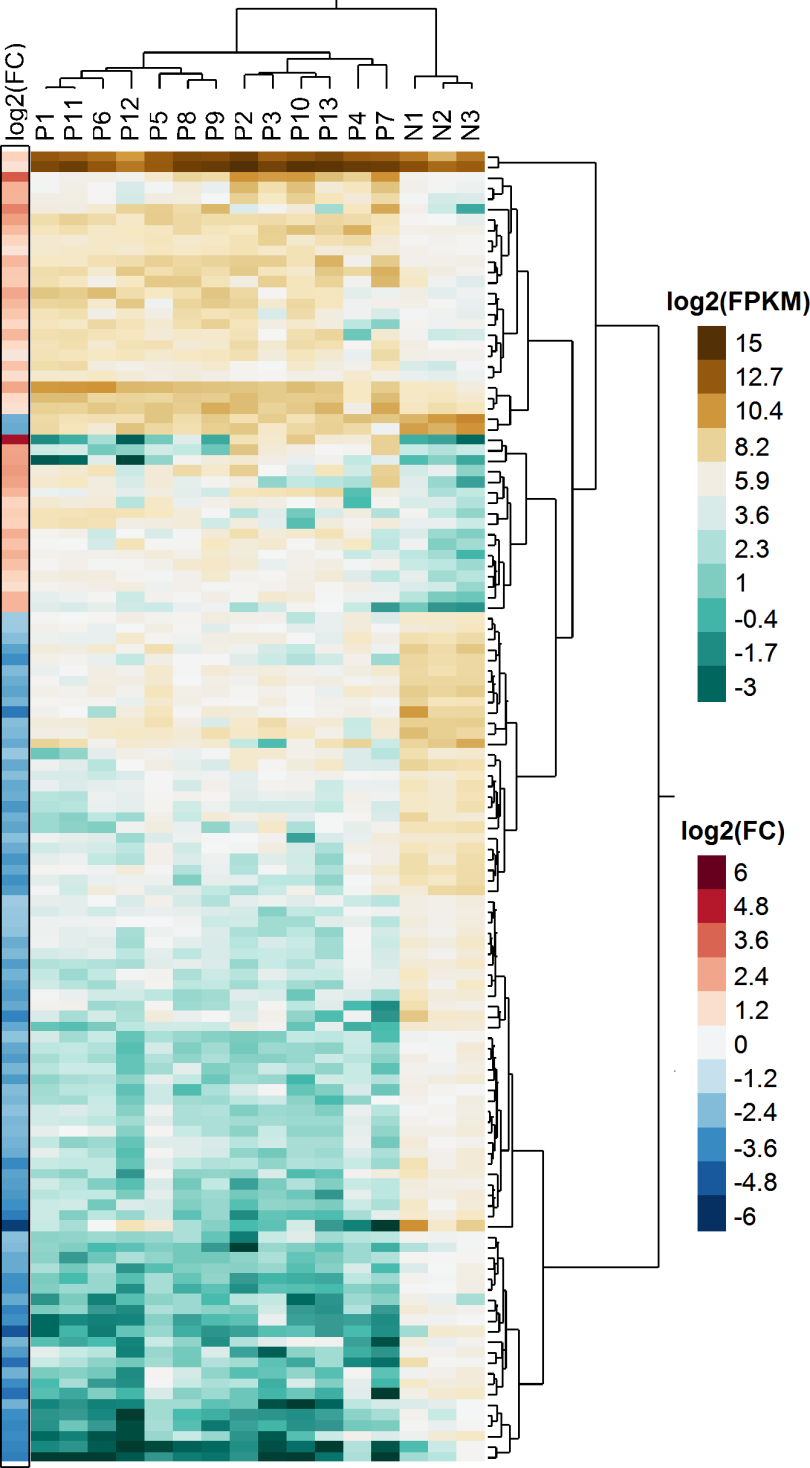
Hierarchical clustering of differentially expressed genes. Consistent gene expression profiles are shown across patient and control groups for log2-transformed FPKM values (aqua/brown heatmap) for the 125 genes found differentially-expressed between meningioma and normal samples. On the left, the relative expression is shown as the log2-transformed fold-change between patients and controls (red/blue heatmap).

Differential gene expression analysis with cuffdiff2 identified 125 of 24,580 annotated genes as significantly (q<0.05) differentially expressed in meningiomas compared to normal meninges (Fig 2). S2 Table lists the 42 genes significantly overexpressed in the meningiomas compared to the controls and S3 Table presents the 83 genes significantly underexpressed in the meningiomas compared to the controls.

Canine chromosome locations were noted for all genes. No genes that would be located on chromosome 22 in the human were differentially expressed. No significantly differentially expressed genes were detected when benign (Grade I) meningiomas were compared to atypical (Grade 2) meningiomas. This is likely due to the small sample sizes and the heterogeneity of the tumors themselves, as is evidenced by the lack of clustering in the principal components analysis.

To investigate global trends in the differential expression signature, the dataset was analyzed for gene set enrichment analysis using the Hallmark gene sets from MSigDB [27, 28]. Gene sets with significant enrichment (S2 Fig, S3 Fig) included several signaling pathways (TNF/NF-kB regulated genes, mTORC1 upregulated genes, Kras up- and down-regulated genes, early and late Estrogen response genes, PI3K/AKT/mTOR upregulated genes, IL2/STAT5 upregulated genes) as well as pathways related to cell cycle (G2M checkpoint, E2F target genes, MYC target genes, apoptosis).

### qPCR

Expression of twelve genes was quantified with qPCR on the same RNA samples to validate the results of the RNAseq transcriptome analysis. Five genes that were down-regulated in the meningiomas compared to the control tissue included AQP1 (aquaporin 1), FRZB (Frizzled b), PECAM1 (platelet and endothelial cell adhesion molecule 1), PDGFRL (platelet-derived growth factor receptor-like), and FBLN2 (fibulin 2). Seven up-regulated genes included MYC (v-myc avian myelocytomatosis viral oncogene homolog), PAMR1 (peptidase domain containing associated with muscle regeneration 1), PDPN (podoplanin), BMPER (BMP binding endothelial regulator), MEDAG (mesenteric estrogen dependent adipogenesis), PERP (TP53 apoptosis effector) and ZC2HC1C (zinc finger C2HC-type containing 1C). Three endogenous control (housekeeping) genes, B2M (beta-2 microglobulin), GUSB (beta-glucuronidase), and RPL13A (ribosomal protein 13A) that were consistently expressed in all samples (S4 Table) were combined to serve as the internal control for each sample. The average of the three normal samples was used for relative expression (reference dCt). Two samples, P1 and P5, were not included in the qPCR analysis because insufficient cDNA was available.

**Fig 3:**
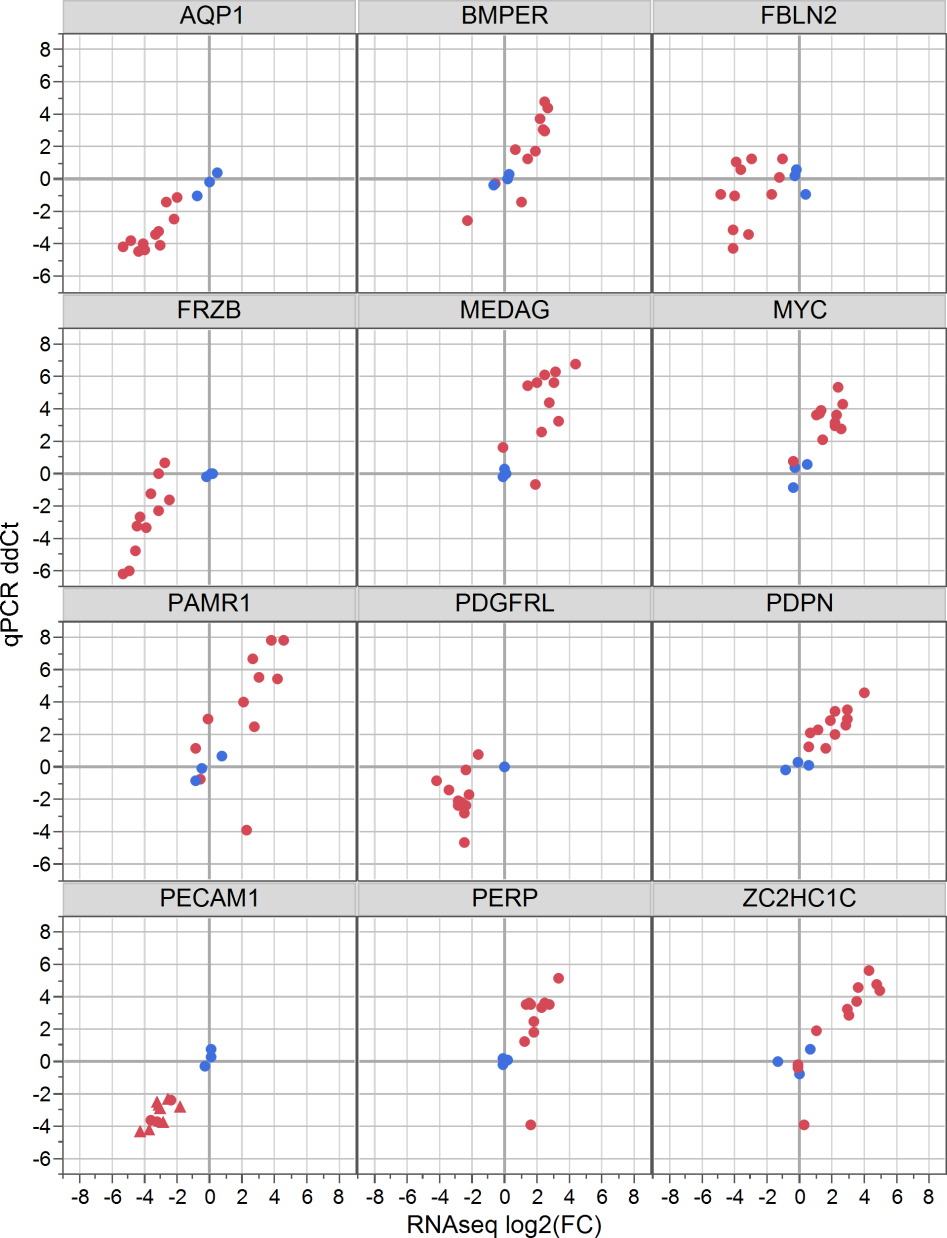
Quantitative PCR for 12 genes found differentially expressed between patients and controls. Validation with qPCR shows that patient samples (red) and normal controls (blue) have similar measurements with both gene expression quantification platforms. X-axis: ddCt values from qPCR: y-axis: log2 (fold-change) values from RNAseq.

The qPCR results were widely congruent with the RNAseq global profiling (Fig 3, S4 Table), demonstrating the validity of the RNAseq measurements. As expected, there was variation in expression among the meningioma samples for these genes. AQP1, FRZB, PDGFRL, and PECAM1 were consistently underexpressed in meningioma samples; MEDAG, MYC, PAMR1, PDPN and PERP were consistently overexpressed in nearly every meningioma sample by both measurements. For these genes, statistical analysis showed that the qPCR results were also significantly different between patients and controls (S4 Table).

## Discussion

In this study we describe the methodology behind successful retrieval and RNAseq transcriptome analysis of RNA from formalin-fixed paraffin embedded canine meningioma samples. This technique has wide application in veterinary and human medicine and we have demonstrated the concept that these tissues can be retrieved for successful differential expression analysis. RNAseq is a valuable modality to identify novel mutations as well as gene expression patterns that may relate to pathogenesis and has proven similarly useful in the study of human meningioma [33]. Reflective of the usefulness and reproducibility of the methods described herein, there were two samples originally included that were removed from the final analysis as they appeared as PCA outliers and their transcriptome revealed genes related to melanocyte function (data not shown). Upon further investigation with the referring veterinarian both of these animals had a previous diagnosis of malignant melanoma and these were diagnosed as melanoma metastasis following transcriptome analysis rather than the original diagnosis of malignant meningioma (also further confirmed with immunohistochemistry). This highlights the usefulness of transcriptome analysis in augmenting and supplementing the basic histologic analysis, as well as the importance of quality checks to remove any low quality or outlier samples that may decrease the statistical power of biological analysis.

The number of clinical meningioma samples studied herein are small and we are therefore unable to make broad characterizations regarding meningioma pathogenesis in the dog; however, the data reveals a number of interesting over and underexpressed genes and global trends indicating shifts in signaling pathways that warrant further investigation. Differential gene expression studies have not commonly been done in canine meningioma; however, previous studies in the dog reported several specific genes that were differentially expressed included those coding for ribosomal proteins, CREG, and TSLC1 among others[34–36].] We have identified a set of 125 differentially expressed (DE) genes in the tumor samples compared to control meninges, which do not include ribosomal protein genes, CREG, or TSLC1. The 42 genes found to be overexpressed in tumors compared to normal meningiomas include a large number of genes previously associated with carcinogenesis and neoplastic transformation. Among the 83 underexpressed genes, there are a number of known tumor suppressor genes, a number of genes associated with angiogenesis (and the PI3K/Akt pathway), as well as genes associated with cell adhesion and the WNT pathway. No NF2 associated alterations were noted which are consistent with those by Thomas et al [8]. In their meningioma panel they did not detect recurrent deletions of the regions of synteny with the human NF2 gene. The NF2 deletion may be associated with alterations in cells leading to the fibroblastic subtype of meningioma [5]. This is consistent with the underrepresentation of this subtype in canine meningioma compared to human meningioma [37,38]. Pavelin et al. [39] also found lower levels of the NF2-associated merlin gene loss in meningothelial meningiomas compared to fibroblastic meningiomas.

Among the 42 overexpressed genes in our study, the majority (23 of 42) are associated with dysregulation of gene expression in one or more human cancers. These include, but are not limited to, FOSB [40], FOS [41], BMPR1B [42], WNT5A [43,44], PDPN [45], BMPER [46], IRF6 [47], and MYC [48,49]. Additionally, FOS, MYC, and PDPN have been shown to be overexpressed specifically in meningeal tumors [41,48,50]. Although there is no evidence linking the expression of the transferrin gene (TF) to overexpression in canine meningiomas, there are reports of the overexpression of the transferrin receptor in human meningiomas [51]. One function of the gene PERP (TP53 apoptosis effector) is the induction of p53 [52], which is overexpressed with MYC in some meningiomas and other cancers.

Six of the 42 genes that are overexpressed are transcription factors. These include EGR1, which is known to bind to the ZFP36 promotor and regulate a number of factors associated with tumor progression in mammary gland tumors, including FOS [53]. An interesting note is that in benign human epithelioid hemangiomas, ZPF36 and FOSB form a fusion product [54]. In the canine genome, these lie in a contingent region of canine chromosome 1 and in our study, a number of meningiomas had increases in both SYNPO2 mRNA complementary to ZPF36 and FOSB. A second zinc finger that is likely also a transcription factor, ZC2HC1C, was noted to be physically close to the FOS gene on canine chromosome 8 and human chromosome 14q24.3. Both transcripts were overexpressed in canine meningiomas compared to the control meninges.

Among the 83 genes underexpressed in canine meningioma compared to control meninges in our study, a number have been identified as tumor suppressors. These include THBS1 [55], COL8A1 [56], FRZB [57], MCAM (CD146) [58], MYCT1 (MTLC) [59], NOTCH3 [60], and PDGFRL [61]. One of the few prognostic indicators for human meningiomas is the loss of 1p (monosomy 1p) which correlates with a loss of expression of ALPL. In our study, ALPL was 4.7 fold underexpressed in meningiomas compared to control meninges. ALPL has been previously recognized as a candidate tumor suppressor gene in meningiomas. [62–64]. There were several underexpressed genes that map to the same region in the canine genome (2:77557722–77614115). These include TINAGL1, COL16A1, PIK3R1, and ECE1, suggesting that this might represent a chromosome deletion.

In addition, several genes underexpressed in the reported cohort are known to be involved in angiogenesis and some are induced by hypoxia and HIF-1-α. GO term enrichment analysis (biological process) reveals overrepresentation of genes involved in angiogenesis [52]. Angiogenesis in tumors is a complex process, with an interplay of many factors and interweaving pathways. For instance, THBS1 is underexpressed in our samples and is known to be an inhibitor of angiogenesis, so its under-expression would be expected to lead to increased angiogenesis and presumably VEGF [65]. THBS1 itself is regulated by BMPER, which we found to be overexpressed.

Other underexpressed genes have roles in cell adhesion. For instance, COL8A1 was identified by microarray analysis to be down-regulated in meningiomas, [56,66]. In addition, in our underexpressed gene cohort, GO enrichment analysis (biological process) identifies a number of genes involved in various aspects of regulation of cell adhesion, cell-cell adhesion, and cell- substrate or -matrix adhesion. Among these are COL16A1, PIK3R1, THBS1, and COL8A1 [25].

Interestingly, another of our underexpressed genes, AMOT, has been found to interact with the Merlin protein (NF2 gene) in the Hippo pathway [67–69]. No evidence of the loss of the canine equivalent of human chromosome 22 has been shown in canine meningiomas. However, this represents the first evidence of dysregulation of a regulator of its gene product.

Six underexpressed genes (SFRP1, FRZB, MCAM, FLT1, CPZ, and DAAM2) have a role in the WNT pathway. SFRP1 has been previously identified as underexpressed in human meningiomas [70]. FRZB is a negative regulator of β-catenin, so a decreased FRZB may be associated with an increase in active beta-catenin. MYC is a target of (the canonical) WNT/β-catenin pathway, so that may underlie the overexpression of MYC found in our study.

Due to small sample size presented herein, there is insufficient information to determine the precise function and interplay of the DE genes and fully appreciate their role in tumorigenesis. There are, however, intriguing interconnections between some genes identified in this study. As an example, WNT5a causes depalmitoylation of MCAM (CD146) which subsequently causes polarization of MCAM and increase in cell mobility. WNT5a also interacts with FLT1 in a pathway referred to as the WNT5a/FLT1 pathway. These interactions suggest that the DE genes identified in our study are not a random set, but reflect the biology of the tumors studied. In the current study there are nearly double the underexpressed genes than overexpressed genes. While the data do not reveal a specific cause for this disparity, tumor differentiation, microenvironmental changes within the tumor, and suppressive effects of overexpressed genes could play a role in the expression differences. Importantly, the gene set enrichment analysis allows for grouping of genes by their interrelatedness and provides further confidence that larger cohorts of canine patients will allow greater elucidation of the molecular fingerprint of canine meningioma and the underlying cellular mechanisms that drive cancer behavior and malignancy.

## Conclusions

We have described a technique that recovers RNA of sufficient quantity for RNAseq transcriptome analysis from formalin-fixed paraffin-embedded canine meningioma samples. Our samples were from standard biopsy cases that are representative of canine meningiomas and gene expression was compared to that in normal canine meninges. We identified a group of 42 significantly overexpressed and 83 significantly underexpressed genes, and identified global trends implicating several signaling pathways. We demonstrated that quantification of the differently expressed genes was reproducible using qPCR technique. Although this was a pilot study in which proving the methodology was the primary objective, we nonetheless have developed a dataset of thirteen animals in which a majority of the genes identified by our analysis are recognized as cancer-associated in humans, thereby reinforcing the view that meningiomas in dogs may have gene expression profiles similar to human meningiomas and be a significant animal model to understand disease pathogenesis, progression, and response to treatment. This study provides good evidence that RNAseq analysis of FFPE tissue is a viable alternative to RNAseq study of fresh-frozen tissue and opens avenues to further study of not just canine meningioma, but other types of canine neoplasia.

## Supporting information

S1 Table Number of reads generated per meningioma sample and mapped to genome and annotated transcriptome. (DOCX)

S2 Table Overexpressed genes in canine meningioma. (DOCX)

S3 Table Underexpressed genes in canine meningioma. (DOCX)

S4 Table Congruency between qPCR analysis and RNAseq profiling. (DOCX)

S1 Fig Analysis of gene body coverage. Minimal 3’ bias is detected in all samples.
(TIF)

S2 Fig Hallmark gene sets enriched in genes upregulated in meningioma samples. Twenty-five Hallmark (MSigDB) gene sets have GSEA FDR (q-value) < 0.05 for genes upregulated in meningioma samples (positive enrichment scores), including several gene sets for signaling pathways. NES = normalized enrichment score; FDR = false discovery rate.
(TIF)

S3 Fig Hallmark gene sets enriched in genes downregulated in meningioma samples. Six Hallmark (MSigDB) gene sets have GSEA FDR (q-value) < 0.05 for genes downregulated in meningioma samples (negative enrichment scores), including genes downregulated by KRAS signalling. NES = normalized enrichment score; FDR = false discovery rate.
(TIF)
