# Supplementary material for: RNA-seq transcriptome analysis of formalin fixed, paraffin-embedded canine meningioma
Source: PLoS One. 2017 Oct 26;12(10):e0187150. doi: 10.1371/journal.pone.0187150 (PMC5658167; doi:10.1371/journal.pone.0187150)
Supplement: S1 Table — (DOCX) [file pone.0187150.s001.docx]

Table S1

|  | **Raw Reads** | **Trimmed/Filtered** | | **Matches rRNA*** | | **Tophat Input** | **Mapped to Genome** | | **Mapped to Annotated Transcriptome** | |
| --- | --- | --- | --- | --- | --- | --- | --- | --- | --- | --- |
| **Sample** | **#** | **#** | **%** | **#** | **%** | **#** | **#** | **%** | **#** | **%** |
| N1 | 41,765,712 | 40,160,695 | 96 | 13,453,994 | 34 | 26,706,701 | 23,973,880 | 90 | 7,400,300 | 31 |
| N2 | 26,515,316 | 20,180,474 | 76 | 2,725,932 | 14 | 17,454,542 | 15,718,455 | 90 | 2,462,133 | 16 |
| N3 | 46,747,229 | 33,426,723 | 72 | 13,810,114 | 41 | 19,616,609 | 17,031,794 | 87 | 4,773,180 | 28 |
| P1 | 27,890,275 | 27,734,794 | 99 | 4,449,878 | 16 | 23,284,916 | 21,096,439 | 91 | 3,595,967 | 17 |
| P2 | 45,653,275 | 43,633,620 | 96 | 5,803,571 | 13 | 37,830,049 | 34,009,761 | 90 | 7,772,722 | 23 |
| P3 | 31,654,248 | 30,625,399 | 97 | 2,932,196 | 10 | 27,693,203 | 24,019,803 | 87 | 4,324,410 | 18 |
| P4 | 32,062,579 | 28,096,043 | 88 | 1,881,723 | 7 | 26,214,320 | 22,672,473 | 86 | 3,741,672 | 17 |
| P5 | 33,589,443 | 28,691,479 | 85 | 6,632,981 | 23 | 22,058,498 | 17,899,864 | 81 | 3,823,683 | 21 |
| P6 | 39,530,633 | 37,430,054 | 95 | 3,617,351 | 10 | 33,812,703 | 30,084,222 | 89 | 5,918,991 | 20 |
| P7 | 25,812,018 | 20,635,552 | 80 | 5,973,275 | 29 | 14,662,277 | 13,184,362 | 90 | 4,376,760 | 33 |
| P8 | 30,175,571 | 29,242,974 | 97 | 1,806,768 | 6 | 27,436,206 | 23,682,901 | 86 | 3,893,760 | 16 |
| P9 | 35,879,371 | 29,686,338 | 83 | 5,608,990 | 19 | 24,077,348 | 20,956,154 | 87 | 6,781,252 | 32 |
| P10 | 32,471,547 | 29,666,312 | 91 | 4,332,841 | 15 | 25,333,471 | 23,697,861 | 94 | 5,097,635 | 22 |
| P11 | 30,255,136 | 27,032,252 | 89 | 7,669,601 | 28 | 19,362,651 | 17,802,093 | 92 | 2,232,395 | 13 |
| P12 | 23,806,802 | 22,695,298 | 95 | 4,815,364 | 21 | 17,879,934 | 16,618,696 | 93 | 7,560,411 | 45 |
| P13 | 26,639,769 | 25,029,969 | 94 | 2,535,118 | 10 | 22,494,851 | 20,118,832 | 89 | 4,555,510 | 23 |
| *avg* | *33,153,045* | *29,622,999* | *90* | *5,503,106* | *18* | *24,119,892* | *21,410,474* | *89* | *4,894,424* | *23* |

*Reads mapped to rRNA and short scRNAs (primarily rRNA)
