# Supplementary material for: RNA-seq transcriptome analysis of formalin fixed, paraffin-embedded canine meningioma
Source: PLoS One. 2017 Oct 26;12(10):e0187150. doi: 10.1371/journal.pone.0187150 (PMC5658167; doi:10.1371/journal.pone.0187150)
Supplement: S2 Table — (DOCX) [file pone.0187150.s002.docx]

Table S2

|  | **Gene symbol** | **Gene description** | **Log2 fold change** | **Ensembl GeneID** |
| --- | --- | --- | --- | --- |
| 1 | FOSB | FosB proto-oncogene, AP-1 transcription factor subunit | 6.0 | ENSCAFG00000004443 |
| 2 | FOS | FBJ murine osteosarcoma viral oncogene homolog | 4.6 | ENSCAFG00000017040 |
| 3 | CTSE | Cathepsin E | 3.8 | ENSCAFG00000010206 |
| 4 | ZC2HC1C | Zinc finger – CysCysHisCys | 3.4 | ENSCAFG00000017024 |
| 5 | KLF5 | Kruppel-like factor 5 | 3.2 | ENSCAFG00000005037 |
| 6 | BMPR1B | Bone morphogenetic protein receptor type 1B | 3.2 | ENSCAFG00000010107 |
| 7 | MYBL1 | MYB proto-oncogene-like 1 | 3.2 | ENSCAFG00000007344 |
| 8 | NR4A1 | Nuclear receptor subfamily 4 group A member 1 | 3.2 | ENSCAFG00000007338 |
| 9 | PAMR1 | Peptidase domain containing associated with muscle regeneration 1 | 3.1 | ENSCAFG00000006865 |
| 10 | GJB2 | Gap junction protein, beta 2 | 3.1 | ENSCAFG00000029537 |
| 11 | GEM | GTP binding protein overexpressed in skeletal muscle | 2.9 | ENSCAFG00000031938 |
| 12 | UPK3B | Uroplakin 3B | 2.9 | ENSCAFG00000013651 |
| 13 | EGR1 | Early growth response 1 | 2.8 | ENSCAFG00000001254 |
| 14 | ZFP36 | Zinc finger protein 36 | 2.8 | ENSCAFG00000005530 |
| 15 | CHGA | Chromogranin A | 2.8 | ENSCAFG00000024864 |
| 16 | NOL4 | Nucleolar protein 4 | 2.8 | ENSCAFG00000017974 |
| 17 | DACT2 | Dishevelled binding antagonist of beta catenin 2 | 2.8 | ENSCAFG00000000866 |
| 18 | AMPD3 | Adenosine monophosphate deaminase 3 | 2.7 | ENSCAFG00000007562 |
| 19 | MEDAG | Mesenteric estrogen-dependent adipogenesis | 2.7 | ENSCAFG00000006579 |
| 20 | KLKB1 | Kallikrein B | 2.7 | ENSCAFG00000007364 |
| 21 | TF | Transferrin | 2.7 | ENSCAFG00000020110 |
| 22 | AMDHD1 | Amidohydrolase domain containing 1 | 2.5 | ENSCAFG00000006406 |
| 23 | GPR133 | G protein-coupled receptor 133 | 2.5 | ENSCAFG00000006655 |
| 24 | WNT5A | Wingless-type MMTV integration site family, member 5A | 2.5 | ENSCAFG00000029321 |
| 25 | RIMS1 | Regulating synaptic membrane exocytosis 1 | 2.4 | ENSCAFG00000002640 |
| 26 | PDPN | Podoplanin | 2.3 | ENSCAFG00000016363 |
| 27 | COMP | Cartilage oligomeric matrix protein | 2.3 | ENSCAFG00000014616 |
| 28 | LPAR3 | Lysophosphatidic acid receptor 3 | 2.2 | ENSCAFG00000020300 |
| 29 | NEAT1_2 | Nuclear paraspeckle assembly transcript 1 | 2.1 | ENSCAFG00000031288 |
| 30 | STXBP6 | Syntaxin binding protein 6 (amisyn) | 2.1 | ENSCAFG00000029607 |
| 31 | MYRF | Myelin Regulatory Factor | 2.1 | ENSCAFG00000016062 |
| 32 | PERP | TP53 apoptosis effector | 2.1 | ENSCAFG00000030892 |
| 33 | BMPER | BMP binding endothelial regulator | 2.1 | ENSCAFG00000003167 |
| 34 | IRF6 | Interferon regulatory factor 6 | 2.0 | ENSCAFG00000011971 |
| 35 | VWA5A | von Willebrand factor A domain containing 5A | 2.0 | ENSCAFG00000011356 |
| 36 | MYC | v-myc myelocytomatosis viral oncogene homolog | 2.0 | ENSCAFG00000001086 |
| 37 | ERMP1 | Endoplasmic reticulum metallopeptidase 1 | 1.9 | ENSCAFG00000002131 |
| 38 | DMXL2 | DMX-like 2 | 1.8 | ENSCAFG00000002131 |
| 39 | WFCD2 | WAP four-disulfide core domain 2 | 1.8 | ENSCAFG00000009696 |
| 40 | NEAT1_1 | Nuclear paraspeckle assembly transcript 1 | 1.7 | ENSCAFG00000029798 |
| 41 | FAM210B | Family With Sequence Similarity 210, Member B | 1.6 | ENSCAFG00000030763 |
| 42 | CTPS1 | CTP Synthase 1 | 1.5 | ENSCAFG00000002732 |
