# Supplementary material for: RNA-seq transcriptome analysis of formalin fixed, paraffin-embedded canine meningioma
Source: PLoS One. 2017 Oct 26;12(10):e0187150. doi: 10.1371/journal.pone.0187150 (PMC5658167; doi:10.1371/journal.pone.0187150)
Supplement: S3 Table — (DOCX) [file pone.0187150.s003.docx]

Table S3

|  | **Gene symbol** | **Gene description** | **Log2 fold change** | **Ensembl GeneID** |
| --- | --- | --- | --- | --- |
| 1 | MYOC | Myocilin | 5.3 | ENSCAFG00000014924 |
| 2 | ALP | Alkaline phosphatase | 4.7 | ENSCAFG00000014853 |
| 3 | CILP2 | Cartilage intermediate layer protein 2 | 4.2 | ENSCAFG00000023249 |
| 4 | COL14A1 | Collagen type XIV alpha 1 chain | 4.1 | ENSCAFG00000000934 |
| 5 | THBS1 | Thrombospondin 1 | 3.9 | ENSCAFG00000008704 |
| 6 | MMRN2 | Multimerin 2 | 3.8 | ENSCAFG00000016086 |
| 7 | ADAMTSL1 | ADAM metallopeptidase with thrombospondin | 3.7 | ENSCAFG00000001581 |
| 8 | PRKD1 | Protein kinase D1 | 3.6 | ENSCAFG00000012553 |
| 9 | COL8A1 | Collagen, type VIII, alpha 1 | 3.6 | ENSCAFG00000031675 |
| 10 | SPTBN5 | Spectrin beta, non-erythrocytic 5 | 3.5 | ENSCAFG00000009849 |
| 11 | FHL5 | Four and a half LIM domains 5 | 3.5 | ENSCAFG00000028858 |
| 12 | MYH11 | Myosin heavy chain 11 | 3.5 | ENSCAFG00000018560 |
| 13 | SFRP1 | Secreted frizzled related protein 1 | 3.4 | ENSCAFG00000005737 |
| 14 | SCN7A | Sodium voltage-gated channel alpha subunit 7 | 3.3 | ENSCAFG00000011648 |
| 15 | ARHGEF15 | Rho guanine nucleotide exchange factor 15 | 3.3 | ENSCAFG00000017057 |
| 16 | FRZB | Frizzled-related protein | 3.3 | ENSCAFG00000024988 |
| 17 | SYNPO2 | Synaptopodin 2 | 3.3 | ENSCAFG00000012424 |
| 18 | ACTC | Actin, alpha 2, smooth muscle | 3.3 | ENSCAFG00000015708 |
| 19 | PTGIR | Prostaglandin I2 receptor | 3.2 | ENSCAFG00000004277 |
| 20 | TIE1 | Tyrosine kinase with immunoglobulin like and EGF like domains 1 | 3.2 | ENSCAFG00000005288 |
| 21 | CD93 | CD93 molecule | 3.2 | ENSCAFG00000005153 |
| 22 | MCAM | Melanoma cell adhesion molecule | 3.1 | ENSCAFG00000012079 |
| 23 | FLT1 | FMS-related tyrosine kinase 1 | 3.0 | ENSCAFG00000006701 |
| 24 | EMCN | Endomucin | 3.0 | ENSCAFG00000032716 |
| 25 | MYCT1 | MYC target 1 | 3.0 | ENSCAFG00000000535 |
| 26 | DAAM2 | Dishevelled associated activator of morphogenesis 2 | 2.9 | ENSCAFG00000001565 |
| 27 | KANK3 | KN motif and ankyrin repeat domains 3 | 2.9 | ENSCAFG00000029570 |
| 28 | LAMC3 | Laminin subunit gamma 3 | 2.9 | ENSCAFG00000019927 |
| 29 | CALCRL | Calcitonin receptor-like | 2.9 | ENSCAFG00000014737 |
| 30 | CPZ | Carboxypeptidase Z | 2.9 | ENSCAFG00000014583 |
| 31 | DCP1/ACE | Angiotensin I converting enzyme | 2.9 | ENSCAFG00000012998 |
| 32 | AQP1 | Aquaporin 1 | 2.8 | ENSCAFG00000003102 |
| 33 | KITLG | KIT ligand | 2.7 | ENSCAFG00000006091 |
| 34 | TINAGL1 | Tubulointerstitial nephritis antigen-like 1 | 2.7 | ENSCAFG00000011002 |
| 35 | PECAM1 | Platelet/endothelial cell adhesion molecule | 2.7 | ENSCAFG00000011740 |
| 36 | CRISPLD2 | Cysteine-rich secretory protein LCCL domain containing 2 | 2.7 | ENSCAFG00000019943 |
| 37 | OLFML3 | Olfactomedin-like 3 | 2.7 | ENSCAFG00000009334 |
| 38 | ESAM | Endothelial cell adhesion molecule | 2.6 | ENSCAFG00000011147 |
| 39 | ADCY2 | Adenylate cyclase 2 | 2.6 | ENSCAFG00000010238 |
| 40 | NOTCH3 | Transmembrane protein | 2.6 | ENSCAFG00000016107 |
| 41 | FBLN2 | Fibulin 2 | 2.6 | ENSCAFG00000004406 |
| 42 | FAM180A | Family with sequence similarity 180, member A | 2.6 | ENSCAFG00000031014 |
| 43 | STC2 | Stanniocalcin 2 | 2.6 | ENSCAFG00000031727 |
| 44 | APOD | Apolipoprotein D | 2.6 | ENSCAFG00000013368 |
| 45 | PTCH2 | Patched 2 | 2.6 | ENSCAFG00000004694 |
| 46 | APOE | Apolipoprotein E | 2.6 | ENSCAFG00000004617 |
| 47 | CACNA2D2 | Calcium voltage-gated channel auxiliary subunit α2Δ2 | 2.5 | ENSCAFG00000010431 |
| 48 | PTPRB | Protein Tyrosine Phosphatase, Receptor Type, B | 2.5 | ENSCAFG00000000446 |
| 49 | FAM198B | Family with sequence similarity 198, member B | 2.5 | ENSCAFG00000032756 |
| 50 | MGLL | Monoglyceride lipase | 2.5 | ENSCAFG00000004093 |
| 51 | PDGFRL | Platelet-derived growth factor receptor-like | 2.5 | ENSCAFG00000006953 |
| 52 | TEK | TEK tyrosine kinase, endothelial | 2.5 | ENSCAFG00000001713 |
| 53 | IGFBP6 | Insulin-Like Growth Factor Binding Protein 6 | 2.5 | ENSCAFG00000028636 |
| 54 | GAS6 | Growth arrest-specific 6 | 2.4 | ENSCAFG00000006421 |
| 55 | ABCA8 | ATP binding cassette subfamily A member 8 | 2.4 | ENSCAFG00000024916 |
| 56 | TLL1 | Tolloid like 1 | 2.4 | ENSCAFG00000008883 |
| 57 | COL16A1 | Collagen, type XVI, alpha 1 | 2.4 | ENSCAFG00000010930 |
| 58 | PALD1 | Phosphatase domain containing, paladin 1 | 2.3 | ENSCAFG00000014067 |
| 59 | PIK3R1 | Phosphoinositide-3-Kinase Regulatory Subunit 1 | 2.3 | ENSCAFG00000007626 |
| 60 | FMN2 | Formin 2 | 2.3 | ENSCAFG00000015625 |
| 61 | MCF2L | MCF.2 cell line derived transforming sequence-like | 2.3 | ENSCAFG00000006240 |
| 62 | NOS3 | Nitric oxide synthase 3 | 2.3 | ENSCAFG00000004687 |
| 63 | ARAP3 | ArfGAP with RhoGAP domain, ankyrin repeat and PH domain 3 | 2.2 | ENSCAFG00000006214 |
| 64 | FMOD | Fibromodulin | 2.2 | ENSCAFG00000009459 |
| 65 | DYSF | Dysferlin | 2.2 | ENSCAFG00000009033 |
| 66 | CYYR1 | Cysteine/tyrosine-rich 1 | 2.2 | ENSCAFG00000031264 |
| 67 | PLVAP | Plasmalemma vesicle associated protein | 2.2 | ENSCAFG00000015337 |
| 68 | DRP2 | Dystrophin related protein 2 | 2.1 | ENSCAFG00000017618 |
| 69 | ZFHX4 | Zinc finger homeobox 4 | 2.1 | ENSCAFG00000008310 |
| 70 | ECE1 | Endothelin converting enzyme 1 | 2.1 | ENSCAFG00000014920 |
| 71 | GM2A | GM2 ganglioside activator | 2.1 | ENSCAFG00000017907 |
| 72 | RCN3 | Reticulocalbin-3 | 2.1 | ENSCAFG00000003620 |
| 73 | AMOT | Angiomotin | 2.0 | ENSCAFG00000018218 |
| 74 | LEPREL2 | Leprecan-like 2 | 2.0 | ENSCAFG00000031318 |
| 75 | TM4SF18 | Transmembrane 4 L six family member 18 | 2.0 | ENSCAFG00000008325 |
| 76 | KALRN | Kalirin, RhoGEF kinase | 1.9 | ENSCAFG00000012281 |
| 77 | PREX2 | Phosphatidylinositol-3,4,5-trisphosphate dependent Rac exchange factor 2 | 1.9 | ENSCAFG00000007620 |
| 78 | HECW | HECT, C2 and WW domain containing E3 ubiquitin protein ligase 2 | 1.8 | ENSCAFG00000010673 |
| 79 | CCDC80 | Ed-coil domain containing 80 | 1.8 | ENSCAFG00000010530 |
| 80 | LIMCH1 | LIM and calponin homology domains 1 | 1.8 | ENSCAFG00000001792 |
| 81 | ETS1 | ETS proto-oncogene 1, transcription factor | 1.7 | ENSCAFG00000010304 |
| 82 | MYO1E | Myosin 1E | 1.6 | ENSCAFG00000016635 |
| 83 | SHE | Src homology 2 domain containing E | 1.6 | ENSCAFG00000017182 |
