# Supplementary material for: RNA-seq transcriptome analysis of formalin fixed, paraffin-embedded canine meningioma
Source: PLoS One. 2017 Oct 26;12(10):e0187150. doi: 10.1371/journal.pone.0187150 (PMC5658167; doi:10.1371/journal.pone.0187150)
Supplement: S4 Table — (DOCX) [file pone.0187150.s004.docx]

Table S4

|  | AQP1 | | BMPER | | FBLN2 | | FRZB | | MEDAG | | MYC | | PAMR1 | | PDGFRL | | PDPN | | PECAM1 | | PERP | | ZC2HC1C | | B2M | GUSB | RPL13A |
| --- | --- | --- | --- | --- | --- | --- | --- | --- | --- | --- | --- | --- | --- | --- | --- | --- | --- | --- | --- | --- | --- | --- | --- | --- | --- | --- | --- |
| Sample | qPCR | RNAseq | qPCR | RNAseq | qPCR | RNAseq | qPCR | RNAseq | qPCR | RNAseq | qPCR | RNAseq | qPCR | RNAseq | qPCR | RNAseq | qPCR | RNAseq | qPCR | RNAseq | qPCR | RNAseq | qPCR | RNAseq | avgCt | avgCt | avgCt |
| N1 | -0.16 (27.0) | 0.05 (180) | 0.01 (31.3) | 0.18 (7) | -0.90 (30.8) | 0.39 (142) | 0.02 (26.5) | 0.15 (124) | -0.21 (29.1) | -0.08 (20) | 0.53 (29.2) | 0.51 (25) | 0.69 (28.1) | 0.78 (25) | 0.06 (25.6) | 0.00 (129) | 0.15 (28.6) | 0.57 (45) | 0.31 (25.8) | 0.12 (188) | -0.19 (28.7) | -0.08 (15) | -0.74 (35.4) | 0.03 (6) | 23.7 | 28.6 | 23.5 |
| N2 | -0.98 (29.0) | -0.78 (101) | 0.31 (32.1) | 0.29 (7) | 0.61 (30.4) | -0.16 (97) | -0.15 (27.8) | -0.23 (95) | 0.25 (29.8) | 0.01 (21) | -0.85 (31.7) | -0.40 (13) | -0.83 (30.8) | -0.84 (8) | 0.01 (26.8) | 0.02 (131) | 0.32 (29.6) | -0.07 (29) | -0.29 (27.6) | -0.29 (141) | 0.05 (29.6) | 0.15 (18) | 0.79 (35.1) | 0.67 (9) | 25.8 | 28.9 | 24.5 |
| N3 | 0.41 (26.6) | 0.47 (242) | -0.32 (31.7) | -0.62 (4) | 0.28 (29.7) | -0.33 (87) | -0.01 (26.6) | 0.05 (116) | -0.05 (29.1) | 0.07 (22) | 0.37 (29.5) | -0.30 (14) | -0.05 (28.9) | -0.46 (11) | 0.08 (25.7) | -0.02 (128) | -0.10 (29.0) | -0.83 (17) | 0.73 (25.5) | 0.13 (189) | 0.14 (28.5) | -0.08 (15) | -0.05 (34.8) | -1.33 (2) | 24.5 | 27.8 | 23.8 |
| P2 | -4.37 (35.8) | -4.02 (11) | 2.99 (32.9) | 2.50 (33) | 0.17 (34.3) | -1.20 (47) | -3.38 (34.5) | -3.87 (8) | 5.62 (27.9) | 3.06 (175) | 4.27 (30.1) | 2.67 (111) | 5.58 (27.8) | 3.01 (117) | -2.09 (32.3) | -2.83 (18) | 2.66 (30.7) | 2.87 (221) | -4.27 (35.0) | -3.66 (14) | 3.38 (29.8) | 2.26 (77) | 4.39 (34.9) | 4.95 (183) | 30.9 | 30.8 | 27.9 |
| P3 | -3.80 (34.9) | -4.85 (6) | 3.79 (31.7) | 2.19 (27) | 1.29 (32.8) | -2.96 (14) | -3.22 (33.9) | -4.42 (5) | 6.32 (26.8) | 3.10 (180) | 3.92 (30.0) | 1.31 (43) | 3.00 (30.0) | -0.08 (14) | 0.81 (29.0) | -1.62 (42) | 2.38 (30.6) | 1.14 (66) | -2.74 (33.1) | -3.15 (19) | 5.13 (27.6) | 3.33 (161) | 1.87 (37.0) | 1.03 (12) | 31.7 | 30.3 | 26.3 |
| P4 | -2.43 (30.7) | -2.21 (38) | -2.55 (35.2) | -2.32 (1) | -0.97 (32.2) | -4.00 (7) | -1.65 (29.5) | -2.49 (20) | 1.65 (28.6) | -0.08 (20) | 0.79 (30.3) | -0.35 (14) | 1.20 (28.9) | -0.90 (8) | -2.35 (29.4) | -2.89 (17) | 2.00 (28.1) | 2.13 (132) | -2.83 (30.3) | -1.83 (49) | 3.50 (26.4) | 2.79 (111) | -0.41 (36.5) | -0.10 (6) | 26.3 | 27.6 | 26.1 |
| P6 | -1.14 (31.5) | -1.99 (44) | 4.47 (30.4) | 2.68 (38) | -0.87 (34.3) | -4.79 (4) | -1.21 (31.3) | -3.56 (9) | 5.64 (26.8) | 2.03 (86) | 3.73 (29.5) | 1.28 (42) | 7.85 (24.5) | 4.58 (348) | -0.12 (29.3) | -2.33 (26) | 1.32 (31.0) | 0.55 (44) | -2.28 (32.0) | -2.54 (30) | 3.58 (28.5) | 1.65 (50) | 4.57 (33.7) | 3.64 (73) | 30.7 | 29.6 | 26.1 |
| P7 | -3.95 (29.2) | -4.06 (10) | 1.25 (28.5) | 1.42 (16) | -4.21 (32.5) | -4.03 (7) | -6.23 (31.2) | -5.30 (3) | 3.24 (24.1) | 3.30 (207) | 3.15 (25.0) | 2.22 (81) | -0.74 (27.9) | -0.54 (10) | -2.10 (26.2) | -2.66 (20) | 4.65 (22.5) | 3.99 (478) | -2.37 (26.9) | -2.33 (34) | 1.19 (25.8) | 1.24 (38) | 4.75 (28.4) | 4.72 (156) | 24.2 | 25.3 | 21.6 |
| P8 | -4.41 (36.3) | -4.37 (8) | 1.88 (34.4) | 0.69 (10) | 0.63 (34.2) | -3.63 (9) | 0.00 (31.5) | -3.15 (13) | 6.09 (27.8) | 2.47 (117) | 3.68 (31.0) | 1.06 (36) | 6.70 (27.0) | 2.64 (91) | -1.38 (32.0) | -3.44 (12) | 3.43 (30.3) | 2.18 (136) | -2.86 (34.0) | -3.04 (21) | 3.64 (29.9) | 1.56 (47) | 5.69 (34.0) | 4.23 (111) | 31.3 | 31.1 | 28.3 |
| P9 | -4.02 (32.6) | -3.00 (22) | 1.79 (31.2) | 1.88 (22) | -3.09 (34.6) | -4.06 (7) | -2.32 (30.5) | -3.13 (13) | 4.41 (26.2) | 2.77 (143) | 2.10 (29.3) | 1.39 (46) | 5.45 (25.0) | 4.15 (258) | -2.81 (30.1) | -2.49 (23) | 1.21 (29.2) | 1.64 (94) | -3.76 (31.6) | -2.82 (24) | 2.51 (27.7) | 1.77 (55) | 2.85 (33.5) | 3.04 (48) | 27.1 | 28.2 | 25.6 |
| P10 | -4.12 (32.1) | -5.26 (5) | -1.40 (33.8) | 1.05 (12) | -0.91 (31.9) | -1.73 (33) | -6.06 (33.7) | -4.91 (4) | -0.65 (30.7) | 1.87 (77) | 2.73 (28.1) | 2.58 (104) | -3.87 (33.7) | 2.25 (69) | -4.60 (31.3) | -2.43 (24) | 2.18 (27.7) | 0.66 (47) | -3.67 (30.9) | -3.55 (15) | -3.95 (33.6) | 1.61 (49) | -3.93 (39.7) | 0.28 (7) | 26.1 | 27.6 | 25.4 |
| P11 | -1.42 (33.4) | -2.67 (27) | 4.77 (31.7) | 2.44 (32) | 1.11 (33.9) | -3.89 (7) | 0.67 (31.0) | -2.72 (17) | 5.49 (28.6) | 1.44 (57) | 5.33 (29.5) | 2.34 (88) | 7.81 (26.1) | 3.76 (196) | -0.84 (31.6) | -4.12 (7) | 2.95 (31.0) | 1.87 (110) | -2.51 (33.8) | -3.25 (18) | 3.56 (30.1) | 1.29 (39) | 3.22 (36.6) | 2.95 (46) | 31.6 | 31.1 | 28.6 |
| P12 | -3.33 (27.4) | -3.34 (17) | -0.28 (28.8) | -0.56 (4) | -3.34 (30.4) | -3.12 (13) | -4.79 (28.5) | -4.59 (5) | 2.59 (23.6) | 2.23 (99) | 3.01 (23.9) | 2.13 (76) | 2.55 (23.4) | 2.72 (96) | -2.37 (25.2) | -2.37 (25) | 2.98 (23.0) | 2.93 (229) | -3.72 (27.1) | -3.24 (18) | 1.81 (24.0) | 1.78 (55) | -0.22 (32.1) | -0.12 (5) | 22.9 | 24.4 | 20.2 |
| P13 | -3.23 (31.8) | -3.16 (20) | 3.05 (29.9) | 2.36 (30) | 1.25 (30.3) | -1.09 (51) | -2.70 (30.9) | -4.26 (6) | 6.79 (23.8) | 4.40 (444) | 3.67 (27.7) | 2.28 (85) | 4.07 (26.4) | 2.13 (64) | -1.65 (29.0) | -2.22 (28) | 3.54 (26.9) | 2.98 (237) | -4.28 (32.1) | -4.27 (9) | 3.63 (26.6) | 2.44 (87) | 3.76 (32.6) | 3.51 (67) | 27.7 | 27.7 | 25.4 |
| **Concordance** |  |  |  |  |  |  |  |  |  |  |  |  |  |  |  |  |  |  |  |  |  |  |  |  |  |  |  |
| ≥2-fold Up | 0 | (0%) | 7 | (64%) | 0 | (0%) | 0 | (0%) | 9 | (82%) | 10 | (91%) | 7 | (64%) | 0 | (0%) | 9 | (82%) | 0 | (0%) | 10 | (91%) | 8 | (73%) |  |  |  |
| ≥2-fold Down | 11 | (100%) | 1 | (9%) | 3 (+3 weak) | (55%) | 9 | (82%) | 0 | (0%) | 0 | (0%) | 0 | (0%) | 8 | (73%) | 0 | (0%) | 11 | (100%) | 0 | (0%) | 0 | (0%) |  |  |  |
| No change | 0 | (0%) | 1 | (9%) | 0 | (0%) | 0 | (0%) | 0 | (0%) | 1 | (9%) | 1 | (9%) | 0 | (0%) | 0 | (0%) | 0 | (0%) | 0 | (0%) | 2 | (18%) |  |  |  |
| Discordant | 0 | (0%) | 2 | (18%) | 5 | (45%) | 2 | (18%) | 2 | (18%) | 0 | (0%) | 3 | (27%) | 3 | (27%) | 3 | (27%) | 0 | (0%) | 1 | (9%) | 1 | (9%) |  |  |  |
| Patients R^2^ | 0.620 | | 0.751 | | 0.149 | | 0.782 | | 0.283 | | 0.388 | | 0.388 | | 0.007 | | 0.627 | | 0.490 | | 0.202 | | 0.782 | |  |  |  |
| REST P(H1) | 0.002 DOWN | | 0.221 |  | 0.546 |  | 0.069 |  | 0.01 UP |  | 0.00 UP |  | 0.02 UP * |  | 0.056 |  | 0.001 UP |  | 0.001 DOWN | | 0.14 |  | 0.001 UP * |  |  |  |  |

qPCR: ddCt (avgCt)

RNAseq: log2FC (FPKM)

* REST analysis of qPCR data (patient vs normal) excluded three samples with < 2-fold change in RNAseq data.
