# Supplementary figures and images for: RNA-seq transcriptome analysis of formalin fixed, paraffin-embedded canine meningioma
Source: PLoS One. 2017 Oct 26;12(10):e0187150. doi: 10.1371/journal.pone.0187150 (PMC5658167; doi:10.1371/journal.pone.0187150)

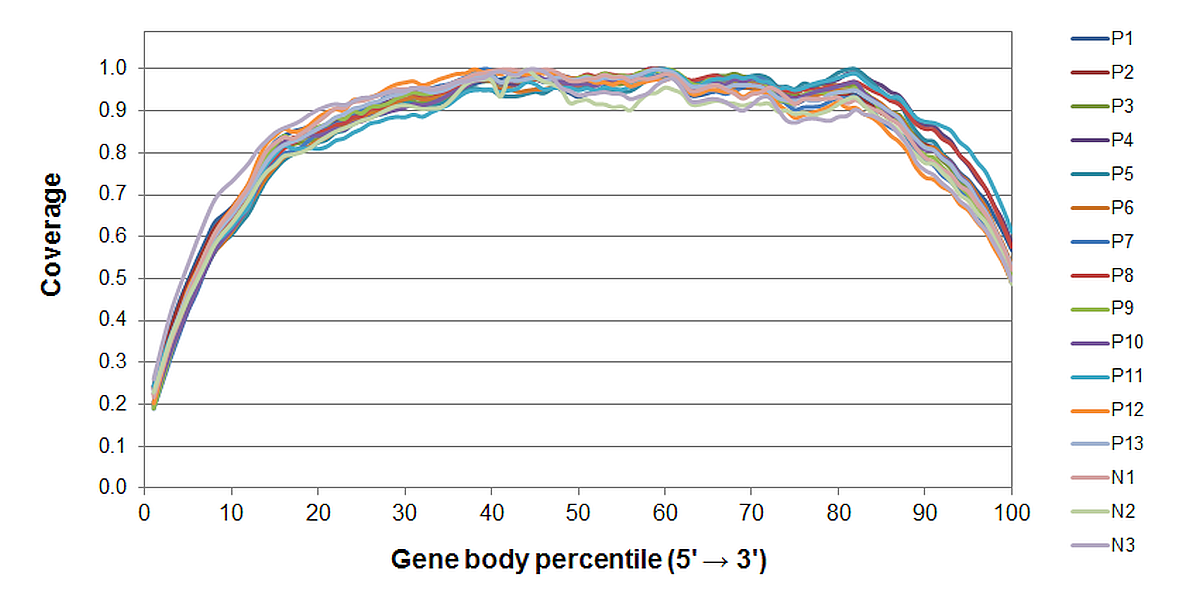

Supplement: S1 Fig — Minimal 3’ bias is detected in all samples. (TIF) [file pone.0187150.s005.tif]

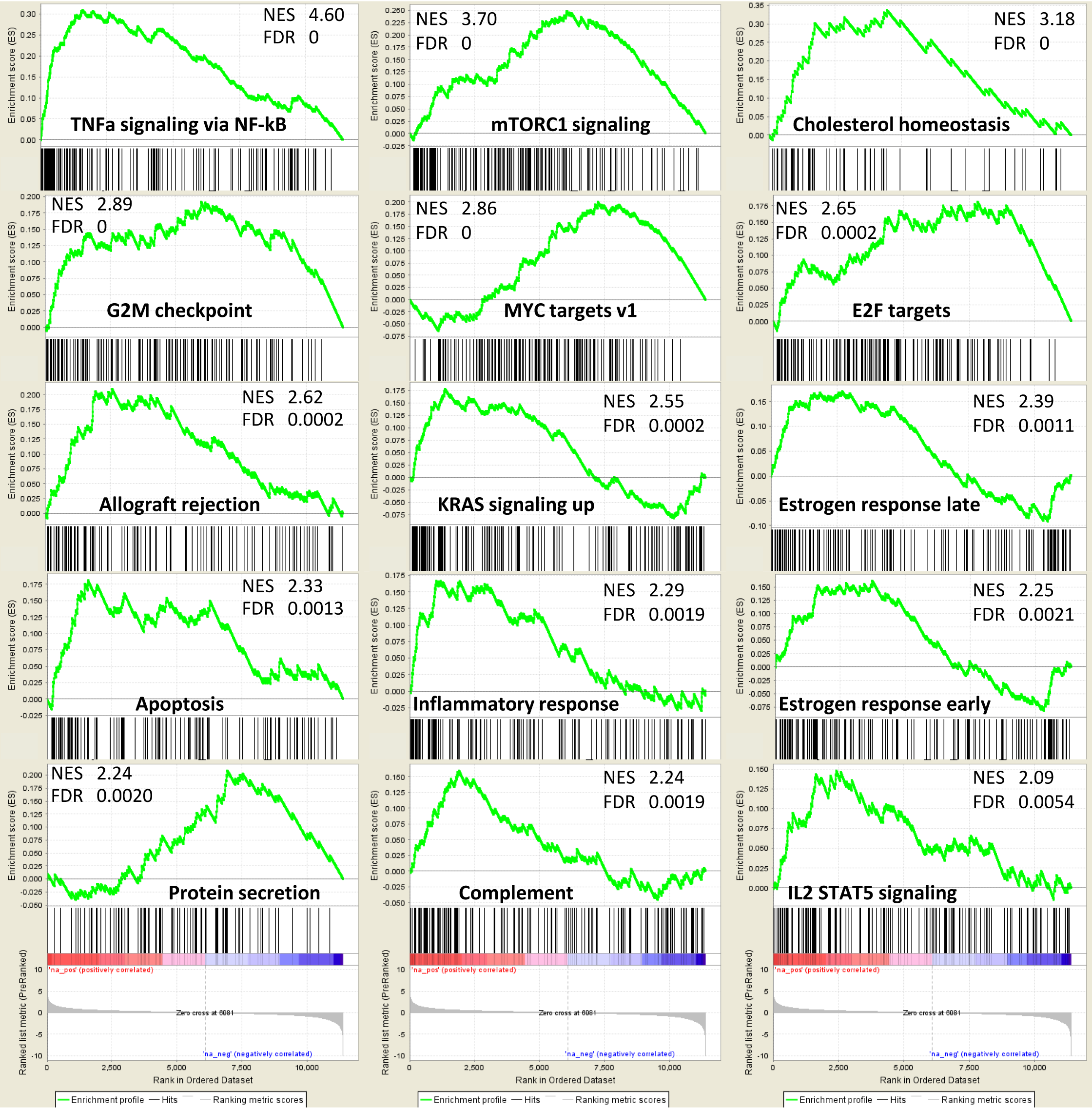

Supplement: S2 Fig — Twenty-five Hallmark (MSigDB) gene sets have GSEA FDR (q-value) < 0.05 for genes upregulated in meningioma samples (positive enrichment scores), including several gene sets for signaling pathways. NES = normalized enrichment score; FDR = false discovery rate. (TIF) [file pone.0187150.s006.tif]

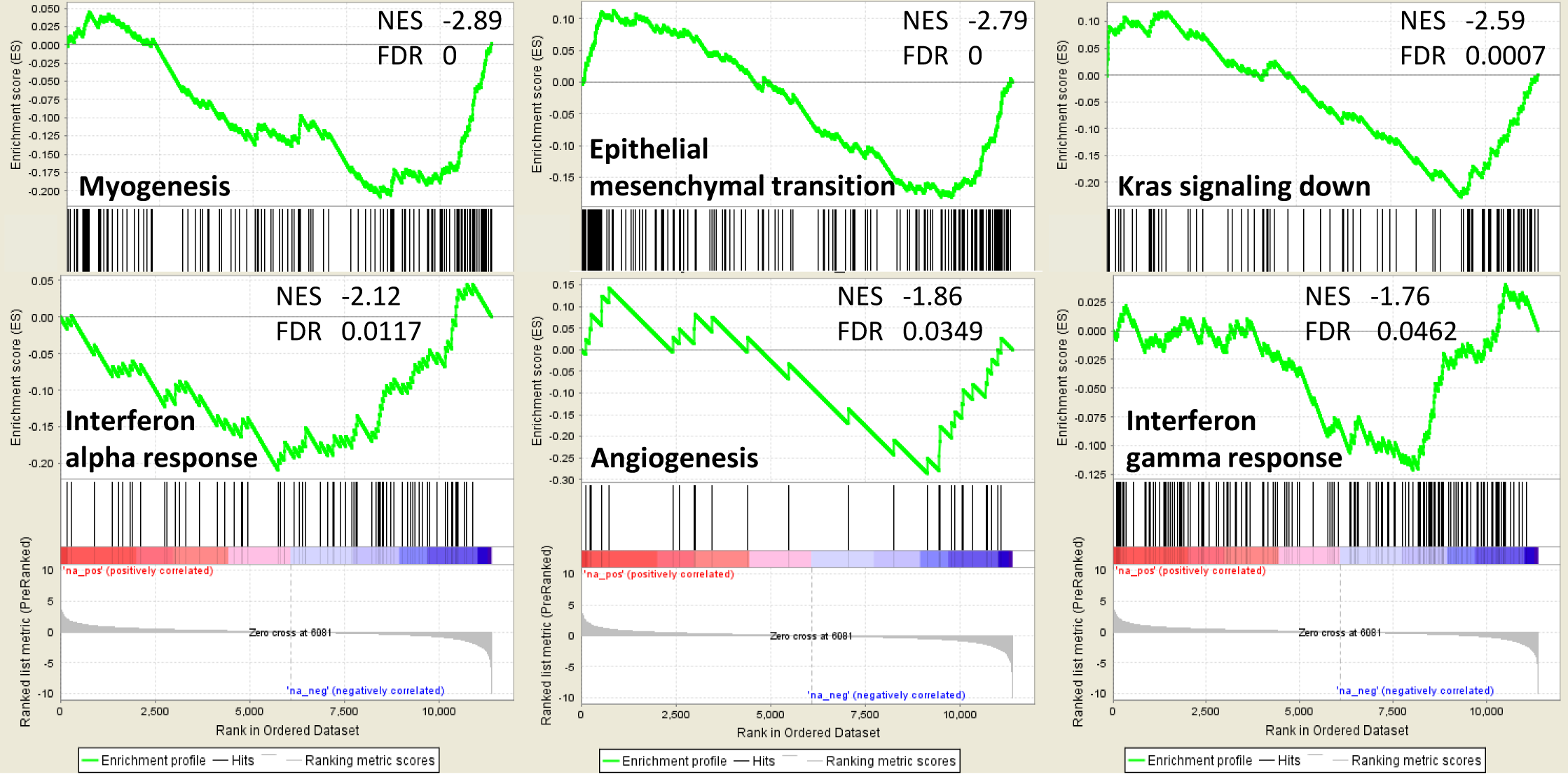

Supplement: S3 Fig — Six Hallmark (MSigDB) gene sets have GSEA FDR (q-value) < 0.05 for genes downregulated in meningioma samples (negative enrichment scores), including genes downregulated by KRAS signalling. NES = normalized enrichment score; FDR = false discovery rate. (TIF) [file pone.0187150.s007.tif]
